# HIF1α/VEGF Feedback Loop Contributes to 5-Fluorouracil Resistance

**DOI:** 10.3389/fphar.2022.851401

**Published:** 2022-03-09

**Authors:** Bin Shi, Fang-Fang Xu, Cai-Ping Xiang, Chuan-Yan Li, Ping Fan, Hao Wang

**Affiliations:** ^1^ Department of Anorectal Surgery, The First Affiliated Hospital of USTC, Division of Life Sciences and Medicine, University of Science and Technology of China, Hefei, China; ^2^ Department of Clinical Laboratory, The First Affiliated Hospital of USTC, Division of Life Sciences and Medicine, University of Science and Technology of China, Hefei, China

**Keywords:** HIF1α, VEGF, EMT, GLUT1, drug resistance, Akt/GSK3β

## Abstract

5-Fluorouracil (5-Fu) is one of the basic drugs in colorectal cancer (CRC) chemotherapy, and its efficacy is mainly limited by the acquisition of drug resistance. However, the underlying mechanisms remain unclear. In this study, hypoxia inducible factor 1α (HIF1α) was screened for high expression in 5-Fu resistant HCT115 cells, which displayed epithelial–mesenchymal transition (EMT) phenotype. Suppression of HIF1α reversed EMT phenotype, reduced glucose transporter 1 (Glut1) expression, a key molecule mediated drug resistance. Moreover, we unveiled that vascular endothelial growth factor (VEGF) was regulated by HIF1α and mediated HIF1α-maintained malignant phenotype of 5-Fu resistant cells. Further studies verified that AKT/GSK3β signaling was activated in resistant cells and controlled HIF1α expression. Interestingly, we demonstrated that VEGF could feedback up-regulate HIF1α via AKT/GSK3β signaling. Clinically, HIF1α and VEGF were high expressed and associated with survival and prognosis in CRC patients. In conclusion, our findings proposed that HIF1α/VEGF feedback loop contributed to 5-Fu resistance, which might be potential therapeutic targets.

## Introduction

Colorectal cancer (CRC) is common malignancy with increasing incidence and leading rate of mortality (Jemal et al., 2011). Despite that the substantial diagnostic and therapeutic strategies have been improving, the mortality rate of CRC remains high (Sun et al., 2020a). 5-Fluorouracil (5-Fu) is a crucial component of conventional chemotherapy for CRC (Jiang et al., 2020). The implementation of 5-Fu-based combination regimens encouraged progress in CRC therapy to date. However, the patients’ response rates to therapy remain low due to the development of drug resistance. Therefore, a better understanding of the molecular mechanisms underlying the chemoresistance will be meaningful for exploring potential strategies to improve the therapeutic outcome.

Hypoxia, controlled by transcription factor hypoxia-inducible factor-1α (HIF-1α), plays important roles in the pathobiology of inflammation and pathology, including regulation of tumor progression (Semenza, 2012). Hypoxia activates the HIF-1α enabling it to trigger transcription and induce an adaptive response (Hong et al., 2020). The role of HIF1α in cancer development extends a broad spectrum of biological functions, such as promoting metastasis, reprograming metabolism, regulating cell proliferation and survival, and increasing therapeutic resistance (Soni and Padwad, 2017). Hypoxic microenvironment mediated-drug resistance has been considered as a major factor of clinical therapy failure.

Emerging evidences have unveiled that the hypoxia-mediated inflammatory microenvironment maintains tumor development and aggressive phenotype (Jiang et al., 2020). Inflammatory cells and cancer cells can secrete proinflammatory cytokines, which facilitate tumor cell motility, angiogenesis, and tissue remodeling, thus promote malignant progression (Candido and Hagemann, 2013; Murata, 2018). The complex interactions between the tumor and inflammatory cells mediated by proinflammatory cytokines are an essential aspect of the hypoxia microenvironment (Eltzschig and Carmeliet, 2011). Vascular endothelial growth factor (VEGF) is a proinflammatory cytokine produced by many kinds of cells. Increasing researches demonstrated that VEGF modulated various crucial processes of tumor development, such as oncogene activation, angiogenesis, metastasis and chemoresistance (Matsumoto and Ema, 2014; Melincovici et al., 2018; Apte et al., 2019).

The molecular mechanisms underlying the development of drug resistance are extremely complicated, including anti-apoptotic, drug efflux, DNA repairment, and glucose metabolism, etc (Long et al., 2019; Abad et al., 2020; Sun et al., 2020b; Wu et al., 2020). Among which, glucose metabolism reprogramming is a cancer hallmark. High rates of glucose metabolism provide ATP energy for cancer cells to biosynthesis (Martinez-Outschoorn et al., 2017). The effectiveness of targeting glucose metabolism approach has been verified by several preclinical researches (Marchiq and Pouyssegur, 2016; Chae and Kim, 2018). Glucose transporter 1 (Glut1) controls glucose transmembrane transportation, which is the first step of glucose metabolism (Wang et al., 2020). In addition to glucose metabolism, high expression of Glut1 protects cancer cells from glucose deprivation-induced oxidative stress and enhances anti-apoptosis activity (Munoz-Pinedo et al., 2012; Gonzalez-Menendez et al., 2018). A recent study proved that suppression of Glut1 inhibited proliferation and glycolysis in enzalutamide-resistant prostate cancer *in vitro* and *in vivo* (Wang et al., 2020).

In the current study, we demonstrate that HIF1α is upregulated in 5-Fu resistant HCT115 cells, and HIF1α maintains resistant phenotype by regulating VEGF/Glut1. We further show that AKT-GSK3β signaling is activated in resistant cells and involved in controlling HIF1α expression. Moreover, VEGF can feedback up-regulate HIF1α via AKT/GSK3β signaling. Therefore, our study suggests an important role of HIF1α/VEGF feedback loop in promoting 5-Fu resistance and provides potential therapeutic targets.

## Materials and Methods

### Chemicals and Reagents

5-Fu was obtained from Sigma-Aldrich (St Louis, MO). LY294002 was obtained from Beyotime Biotechnology (Shanghai, China). Primary antibodies against E-cadherin, Vimentin, ABCB1, ERCC1, p53, LCI-II, Snail, Slug, ZEB1, *p*-GSK-3β (ser9), GSK-3β, *p*-Akt (Ser473), Akt, p-p65, p65, pMAPK38, MAPK38, and HIF1α were obtained from Cell Signaling Technology (MA, United States). Primary antibodies against ABCC3, ABCC10, ABCC12 were obtained from Abcam (Cambridge, United Kingdom). Primary antibodies against β-catenin, Twist, Glut1 were obtained from Santa Cruz Biotechnology (Santa Cruz, CA, United States). Primary antibodies against ABCC1, ABCC2, Bcl-2, PARP, LAT1, KLF12, CA9, BAX, VEGF and GAPDH were purchased from Proteintech (Wuhan, China). HRP-conjugated and Alexa Fluor 594 conjugated secondary antibody, DAPI, Trizol and Lipofectamine 2000 were purchased from Life (CA, United States). PrimeScript^®^ RT reagent Kit and SYBR^®^ Premix Ex Taq^TM^ were products of TaKaRa.

### Cell Culture

The colorectal cancer (CRC) cells HCT15 and HCT116 were obtained from American Type Culture Collection (ATCC). 5-Fu resistant HCT15 cells (HCT15/5-Fu) were derived in our laboratory. Briefly, HCT15 cells were treated with 1 μM 5-Fu for 48 h, then replaced with drug-free culture medium. After the cells recovered, the 1 μM 5-Fu was added again. Repeat this process four times and gradually increase 5-Fu concentration. HCT15/5-Fu cells were cultured in DMEM/F12 culture medium with 1 μM 5-Fu to maintain their resistance. The culture medium was replaced with drug-free 48 h prior to the experiments. HCT15 cells were maintained in DMEM/F12 culture medium supplemented with 10% FBS. The cells were cultured under a humidified 5% CO2 atmosphere at 37°C in incubator.

### RNA Interference and Plasmid Transfection

siRNAs against human Glut1 and HIF1α were purchased from GenePharma (Shanghai, China). The siRNA sequences are listed in Table 1. Empty plasmid pcDNA3.1 was obtained from Promega and expression plasmid for pcDNA-Glut1 was established. 2×10^5^ cells/well were seeded on a 6-well plate and left in culture until the next day. They were then transfected with siRNA oligonucleotides (100 nM) or plasmids (2 mg) mixed with lipofectamine 2000 reagent (Invitrogen) in serum-free medium according to the manufacturer’s instructions. 6 h later, medium was changed to complete culture medium and the cells were incubated for another 24 h before harvest.

**TABLE 1:** siRNA sequences used in the study.

Gene	Sence (5′-3′)	Anti-Sence (5′-3′)
Control siRNA	UUC​UCC​GAA​CGU​GUC​ACG​UTT	ACG​UGA​CAC​GUU​CGG​AGA​ATT
HIF1α siRNA	GAU​GAA​AGA​AUU​ACC​GAA​UTT	AUU​CGG​UAA​UUC​UUU​CAU​CTT
Glut1 siRNA	GCC​CAU​GUA​UGU​GGG​UGA​AGU​GUC​A	UGA​CAC​UUC​ACC​CAC​AUA​CAU​GGG​C

### Transwell Migration Assay

For transwell migration assays, cells (1×10^5^/insert) suspended in 200 μl serum free culture media were seeded into the upper chamber, while complete media was added to the lower chamber. After 24 h of incubation, the cells migrated to the bottom of chamber were fixed in 4% paraformaldehyde for 10 min, stained with hematoxylin and counted (five fields per chamber). The experiments were performed in triple.

### Cell Viability Assay

CCK8 agent was used for cell viability assay. Cells (2×10^4^ cells/well) were seeded in 96-well plates and treated with different concentrations of 5-Fu for 48 h. The 10 μL CCK8 agent was added to each well and the plates were incubated at 37°C for 2 h. The absorbance was measured at 570 nm using a microplate reader. The experiments were performed in triple.

### Glucose Concentration Detection

The glucose concentration in cultured media were measured using commercial kits (BioVision) following the manufacturer’s instructions. Briefly, Cells (2×10^4^ cells/well) were seeded in 96-well plates, differentiated, then maintained for another 4 days. To assay glucose uptake, cells were washed twice with PBS and starved in 100 μl serum free medium overnight, then rewashed twice with PBS. The cells were starved for glucose by preincubating with 100 μl Krebs-Ringer-Phosphate-HEPES (KRPH) buffer containing 2% BSA for 40 min, then stimulated with 1 μM insulin for 20 min to activate glucose transporter. 10 μl 2-Deoxyglucose (2-DG, 10 μM) was added and the cells was incubated for 20 min 10 μl Enzyme Mix reaction was added into each well. Mix and incubate at 37°C for 1 h. 90 μl extraction buffer was added, then the plate was sealed and heated at 37°C for 40 min to degrade unused NADP. The plate was cooled on ice for 5 min and added with 12 μl neutralization buffer, then 38 μl Gluathione reductase and substrate (DTNB) Mix was added to each well. Finally, Glucose concentrations in the supernatants were measured using microplate reader at 412 nm and calculated from standard curve. All samples were tested in triplicate.

### Quantitative Real-Time PCR

Trizol reagent (Invitrogen, United States) was employed to extract total RNA from cells of different treatment groups according to the manufacturer’s guidelines. The mRNA levels of target genes were assessed on ABI-7500 (Carlsbad, California, United States) and were normalized to GAPDH. The relative expression levels for target gene were calculated using the comparative threshold cycle (CT) (2^−ΔΔCT^) method. The primers used are listed in Table 2.

**TABLE 2:** Primers used in the RT-PCR assay.

Primers	Forward primer 5′-3′	Reverse primer 5′-3′
E-cadherin	TAC​ACT​GCC​CAG​GAG​CCA​GA	TGG​CAC​CAG​TGT​CCG​GAT​TA
Vimentin	TGA​GTA​CCG​GAG​ACA​GGT​GCA​G	TAG​CAG​CTT​CAA​CGG​CAA​AGT​TC
Glut1	AAG​ACA​GCG​TTG​ATG​CCA​GAC	GAT​GAT​GCG​GGA​GAA​GAA​GGT
ERCC1	CTG​GAG​GTG​ACC​AAA​CTC​ATC​TA	AGT​GGG​CTT​GGT​TTT​GGT​CTG​G
ABCB1	TGC​TCA​GAC​AGG​ATG​TGA​GTT​G	AAT​TAC​AGC​AAG​CCT​GGA​ACC
ABCC1	GCCAAGAAGGAGGAGACC	AGG​AAG​ATG​CTG​AGG​AAG​G
ABCC2	TGG​TGG​CAA​CCT​GAG​CAT​AGG	ACT​CGT​TTT​GGA​TGG​TCG​TCT​G
ABCC3	CTT​AAG​ACT​TCC​CCT​CAA​CAT​GC	GGT​CAA​GTT​CCT​CTT​GGC​TC
ABCC10	ATT​GCC​CAT​AGG​CTC​AAC​AC	AGC​AGC​CAG​CAC​CTC​TGT​AT
ABCC12	GGT​GTT​CAT​GCT​GGT​GTT​TGG	GCT​CGT​CCA​TAT​CCT​TGG​AA
ABCG2	TAT​AGC​TCA​GAT​CAT​TGT​CAC​AGT​C	GTT​GGT​CGT​CAG​GAA​GAA​GAG
CA9	GTC​CAG​CTG​AAT​TCC​TGC​CT	CCT​TCT​GTG​CTG​CCT​TCT​CA
LAT1	GTG​GCT​GTG​GAT​TTT​GGG​AAC	ATT​CAC​CTT​GAT​GGG​ACG​CTC
Bcl-2	GGT​GAA​CTG​GGG​GAG​GAT​TGT	CTT​CAG​AGA​CAG​CCA​GGA​GAA
P53	GCG​CAC​AGA​GGA​AGA​GAA​TCT​CCG	TTT​GGC​TGG​GGA​GAG​GAG​CTG
PARP	AAG​AAA​TGC​AGC​GAG​AGC​AT	CCA​GTG​TGG​GAC​TTT​TCC​AT
Bax	GGG​ACG​AAC​TGG​ACA​GTA​ACA	CCG​CCA​CAA​AGA​TGG​TCA​C
Slug	TTC​GGA​CCC​ACA​CAT​TAC​CT	GCA​GTG​AGG​GCA​AGA​AAA​AG
Snail	GAC​CAC​TAT​GCC​GCG​CTC​TT	TCG​CTG​TAG​TTA​GGC​TTC​CGA​TT
Twist	GGA​GTC​CGC​AGT​CTT​ACG​AG	TCT​GGA​GGA​CCT​GGT​AGA​GG
ZEB1	TAC​AGA​ACC​CAA​CTT​GAA​CGT​CAC​A	GAT​TAC​ACC​CAG​ACT​GCG​TCA​CA
β-catenin	GCG​TTC​TCC​TCA​GAT​GGT​GTC	CCA​GTA​AGC​CCT​CAC​GAT​GAT
HIF1α	ATG​ACT​CCT​TTT​CCT​GCT​CTG	CTC​CAT​CTC​CTA​CCC​ACA​TAC​A
TGFβ	CAA​TTC​CTG​GCG​ATA​CCT​CAG	AGA​TAA​CCA​CTC​TGG​CGA​GTC
TNFα	TCC​TTC​AGA​CAC​CCT​CAA​CC	AGG​CCC​CAG​TTT​GAA​TTC​TT
VEGF	AGC​CTT​GCC​TTG​CTG​CTC​TA	GTGCTGGCCTTGGTGAGG
EGF	GGT​CTT​GCT​GTG​GAC​TGG​AT	CTG​CTA​CAG​CAA​ATG​GGT​GA
PDGF	GCA​AGA​CCA​GGA​CGG​TCA​TTT	GGC​ACT​TGA​CAC​TGC​TCG​T
b-FGF	ACC​CTC​ACA​TCA​AGC​TAC​AAC	AAA​AGA​AAC​ACT​CAT​CCG​TAA
HGF	GCC​TGA​AAG​ATA​TCC​CGA​CA	GCC​ATT​CCC​ACG​ATA​ACA​AT
IGF	TCA​CCT​TCA​CCA​GCT​CTG​C	TGG​TAG​ATG​GGG​GCT​GAT​AC
GAPDH	GCACCGTCAAGGCTGAGAACAC	TGGTGAAGACGCCAGTGGA

### Western Blotting

The total proteins were extracted from cells with WB and IP lysis buffer (Beyotime Biotechnology, Shanghai, China). Equal amounts of proteins were loaded in SDS-polyacrylamide gels, and transferred to PVDF membranes (Millipore, USA). After blocking with 5% BSA at room temperature for 2 h, the membranes were incubated with primary antibodies at 4°C overnight and then incubated with secondary antibodies for 1.5 h at room temperature. Proteins were then measured by an enhanced chemiluminescence system (ECL) reagent (KeyGEN BioTECH, China). Band intensity was quantified by densitometry analysis using Image-Pro Plus 4.5 software (Rockville, MD, United States).

### Immunofluorescence

The cells were fixed with 4% paraformaldehyde for 20 min and permeabilized with 0.5% Triton X-100 for 15 min. After blocked with goat serum for 1.5 h at room temperature, the cells were incubated with primary antibodies against E-cadherin, Vimentin or HIF1α (1:100 dilution) at 4ºC overnight. Following incubated with Alexa Fluor 594-conjugated secondary antibody (1:1,000 dilution) for 1 h at room temperature, the cells were mounted with DAPI (10 μg/ml) for 10 min. The images were obtained with Confocal Laser Scanning Microscopy (Zeiss).

### Nuclear/Cytoplasm Separation

The nuclear and cytoplasm proteins from cells were obtained by using a nuclear/cytosol fractionation kit (BioVision), and western blotting analysis was performed as described above.

### Immunohistochemistry

Sixty pairs of CRC tumor tissues and adjacent normal tissues were collected from The First Affiliated Hospital of USTC. The tissues were collected, paraffin embedded, and cut into sections. The sections were incubated with 10% goat serum for 2 h at room temperature, anti-HIF1α antibody (1:200 dilution), anti-VEGF antibody (1:200 dilution) at 4°C overnight, secondary antibody for 1.5 h at room temperature, respectively. DAB was used as substrate and Mayer’s hematoxylin was applied as counterstain. The images were obtained form an inverted microscope (Nikon, Japan). The intensity of staining of HIF1α and VEGF were independently evaluated by two pathologists in the following four categories: no staining = 0, weak staining = 1, moderate staining = 2, and strong staining = 3. The stain-positive sections were categorized into four grades: 0 (0%), 1 (1%–33%), 2 (34%–66%), and 3 (67%–100%). The final score was calculated by multiplying the percentage of positive cells with the intensity score.

### Statistical Analysis

Results were expressed as Mean ± SD of three independent experiments unless otherwise specified. Data were analyzed by Student’s t-test between any two groups. One-way ANOVA analysis of variance was used to assess the difference of means among groups. Pearson’s χ2 test was used to measure the expression difference of HIF1α in CRC tumor samples and adjacent normal tissues. Kaplan-Meier method was used to evaluate survival curves. These analyses were performed using GraphPad Prism Software Version 5.0 (GraphPad Software Inc., La Jolla, CA). A P-value of <0.05 was considered statistically significant.

## Results

### 5-Fu Resistant HCT15 Cells Acquire the EMT Phenotype

**FIGURE 1:**
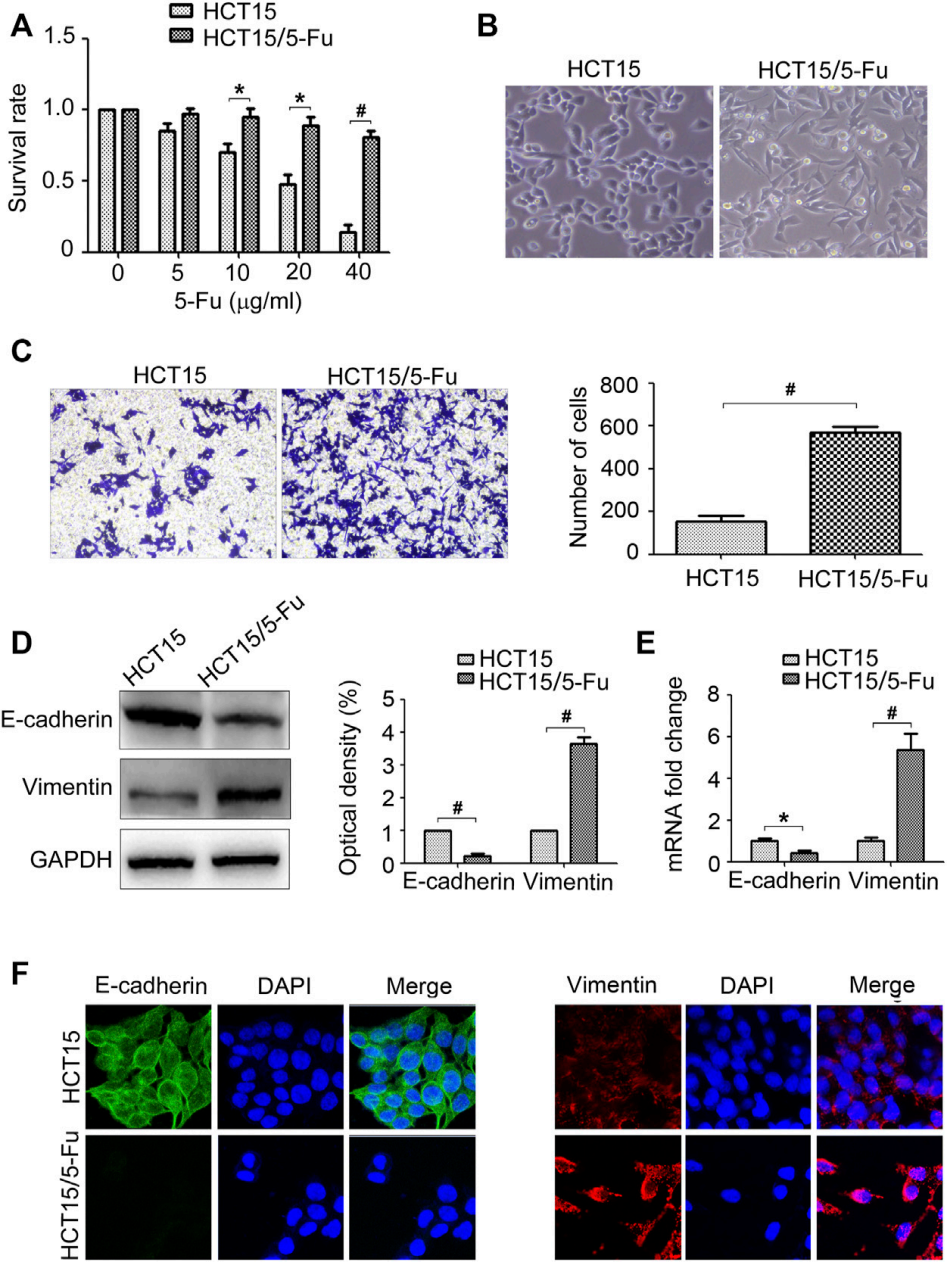
5-Fu resistant CRC cells acquire EMT phenotype. **(A)** HCT15 and HCT15/5-Fu cells were treated with increasing concentrations of 5-Fu for 48 h. CCK8 assay was used to quantify the viable cells. **(B)** Morphology of HCT15 and HCT15/5-Fu cells. **(C)** Transwell assay was performed to measure the migration of HCT15 and HCT15/5-Fu cells. **(D,E)** The protein and mRNA expression of E-cadherin and Vimentin in HCT15 and HCT15/5-Fu cells were examined by WB and RT-PCR, respectively. **(F)** The expression and cellular localization of E-cadherin and Vimentin were detected by IF staining. Nuclei were visualized with DAPI staining. Data represented as mean ± SD were from three independent experiments. *: *p* < 0.05, #: *p* < 0.01.

Firstly, the 5-Fu resistant phenotype of HCT15/5-Fu cells was verified. The results of CCK8 assay showed that HCT15/5-Fu cells displayed 5-Fu resistance compared with its parent cells (Figure 1A). In term of the morphology, HCT15 cells displayed a typical cobblestone epithelial feature. On the contrary, HCT15/5-Fu cells exhibited a spindle mesenchymal appearance (Figure 1B). Morphological changes reminded that HCT15/5-Fu cells acquired EMT phenotype. The transwell assay showed that the migration capability of HCT15/5-Fu cells was significantly increased compared with HCT15 cells (Figure 1C). As illustrated by WB and RT-PCR assay, the epithelial maker E-cadherin was down-regulated, while the mesenchymal markers Vimentin was up-regulated in HCT15/5-Fu cells (Figures 1D,E). IF staining further confirmed this phenomenon (Figure 1F). Collectedly, these results indicated that HCT15/5-Fu cells acquired EMT characteristic.

### Glut1 Controls the 5-Fu Resistant Phenotype of HCT15/5-Fu Cells

**FIGURE 2:**
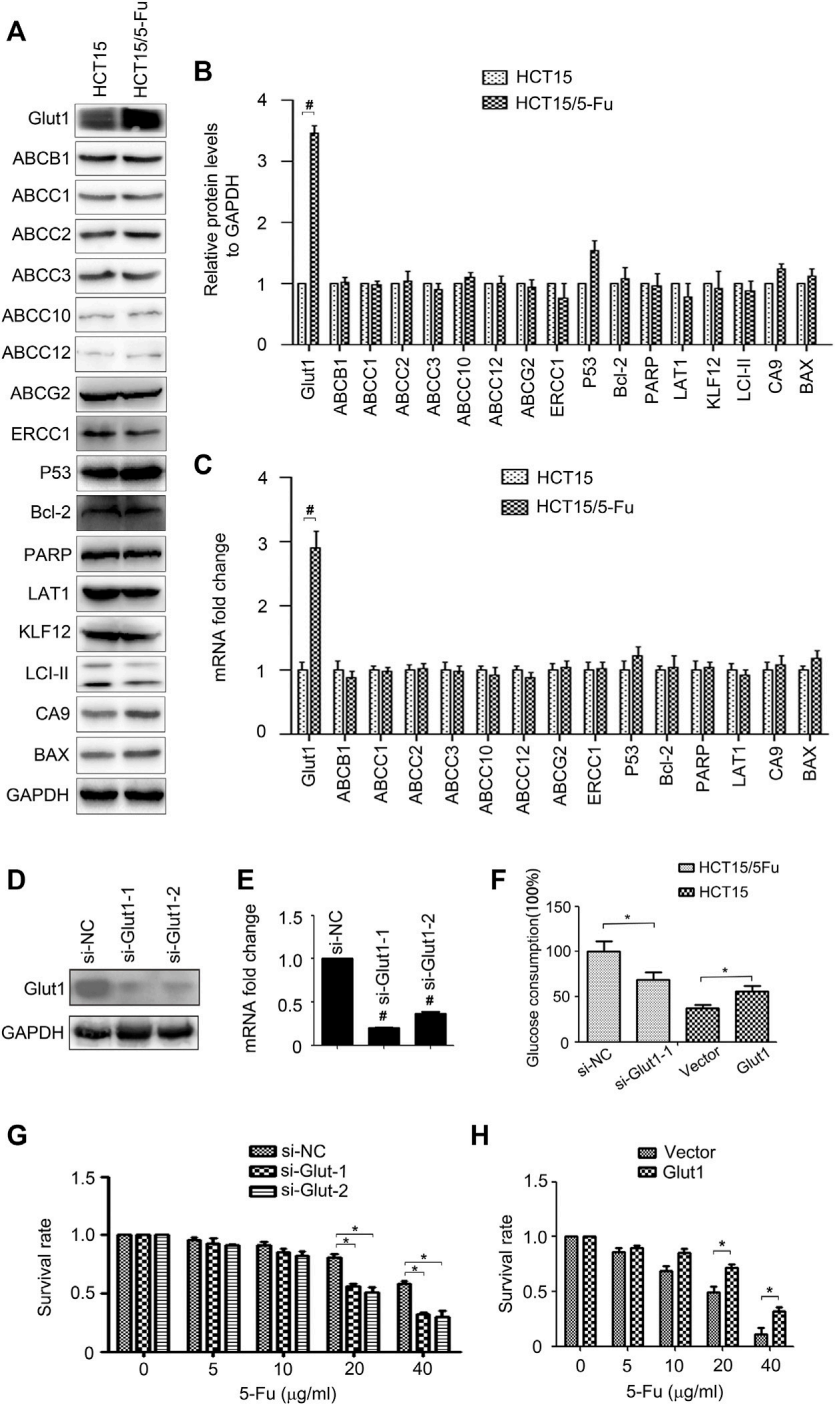
Glut1 maintains the resistance phenotype of 5-Fu resistant CRC cells. **(A,B)** The protein expression of Glut1, ABCB1, ABCC1, ABCC2, ABCC3, ABCC10, ABCC12, ABCG2, ERCC1, P53, Bcl-2, PARP, LAT1, KLF12, LCI-II, CA9 and BAX in HCT15 and HCT15/5-Fu cells were examined by WB. GAPDH servers as the loading control. **(C)** The mRNA expression of Glut1, ABCB1, ABCC1, ABCC2, ABCC3, ABCC10, ABCC12, ABCG2, ERCC1, P53, Bcl-2, PARP, LAT1, CA9 and BAX in in HCT15 and HCT15/5-Fu cells were examined by RT-PCR. **(D,E)** Expression of Glut1 protein and mRNA in HCT15/5-Fu cells transfected with si-NC or si-Glut1 for 24 h were detected by WB and quantitative RT-PCR, respectively. **(F)** Glucose concentration in the supernatants was measured in HCT15/5-Fu cells transfected with si-NC or si-Glut1 and HCT15 cells transfected with control vector plasmid or pcDNA-Glut1. **(G)** HCT15/5-Fu cells were transfected with si-NC or si-Glut1 for 24 h were treated with increasing concentrations of 5-Fu for 48 h. CCK8 assay was used to quantify the viable cells. **(H)** HCT15 cells were transfected with control vector plasmid or pcDNA3.1-Glut1 for 24 h were treated with increasing concentrations of 5-Fu for 48 h. CCK8 assay was used to quantify the viable cells. Data represented as mean ± SD were from three independent experiments. *: *p* < 0.05, #: *p* < 0.01. si-NC: negative control siRNA.

To investigate the molecular mechanism underlying 5-Fu resistance, the expressions of drug resistant relative genes, including Glut1, ABCB1, ABCC1/2/3/10/11/12, ABCG2, ERCC1, P53, Bcl-2, PARP, LAT1, KLF12, LCI-II, CA9 and BAX were determined by WB and RT-PCR assay, respectively. The results found that the expressions of Glut1 mRNA and protein were increased, but not the others (Figures 2A–C). To validate the role of Glut1 in regulation of 5-Fu resistance, si-Glut1s were used. As shown in Figures 2D,E, Glut1 was distinctly inhibited in HCT15/5-Fu cells. Glut1 knockdown decreased glucose consumption of HCT15/5-Fu cells while overexpression Glut1 increased glucose consumption of HCT15 cells (Figure 2F). Importantly, inhibition of Glut1 decreased 5-Fu resistance of HCT15/5-Fu cells (Figure 2G). Contrarily, Glut1 overexpression promoted 5-Fu resistance of HCT15 cells (Figure 2H). These results indicated that Glut1 was critical for 5-Fu resistant phenotype of HCT15/5-Fu cells.

### HIF1α is Upregulated in 5-Fu Resistant HCT15 Cells

**FIGURE 3:**
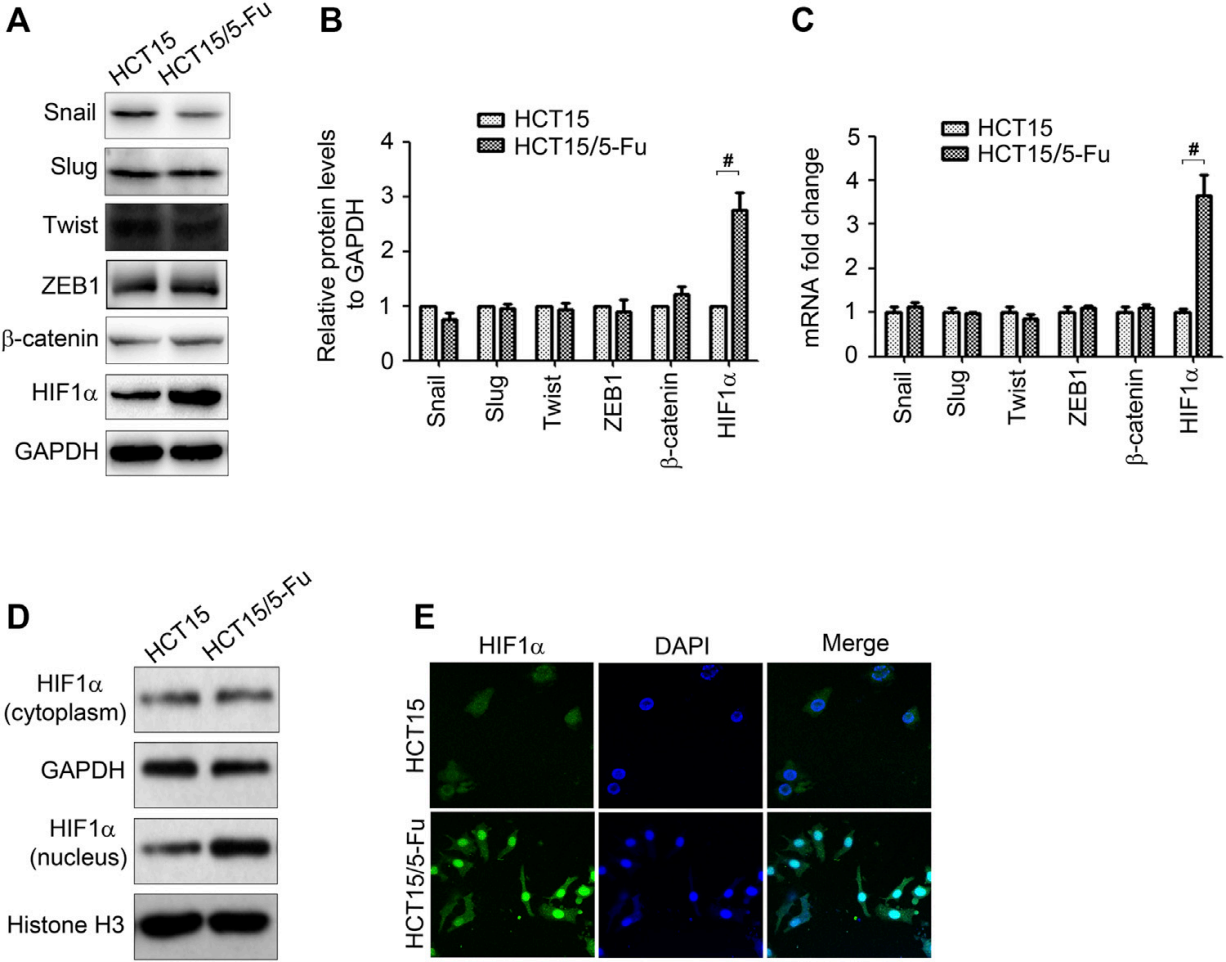
HIF1α is up-regulated in 5-Fu resistant CRC cells. **(A–C)** The protein and mRNA expression of Snail, Slug, Twist, ZEB1, β-catenin and HIF1α in HCT15 and HCT15/5-Fu cells were examined by WB and RT-PCR, respectively. **(D)** The HIF1α expression in cytoplasm and nucleus in HCT15 and HCT15/5-Fu cells were examined by WB. **(E)** The cellar localization of HIF1α in HCT15 and HCT15/5-Fu cells was analyzed by IF staining. Nuclei were visualized with DAPI staining. #: *p* < 0.01.

Several transcript factors play important role in controlling EMT conversion, such as Snail, Slug, Twist, ZEB1, and β-catenin (Peinado et al., 2007). However, according to WB and RT-PCR results, the expression of these transcript factors have no obvious change in HCT15/5-Fu cells (Figures 3A–C). We next determined HIF1α expression since it is an upstream of Glut1 and is involved in regulation of EMT. Surprisingly, HIF1α mRNA and protein expression both increased (Figures 3A–C). As a transcription factor, HIF1α usually exerts its function in nucleus. We separated the cytoplasm and nucleus of cells and measured HIF1α expression. As shown in Figure 3D, HIF1α expression in nucleus was upregulated in HCT15/5-Fu cells compared with HCT15 cells. The images from laser scanning confocal microscope further confirmed this conclusion (Figure 3E).

### HIF1α Regulates VEGF Expression

**FIGURE 4:**
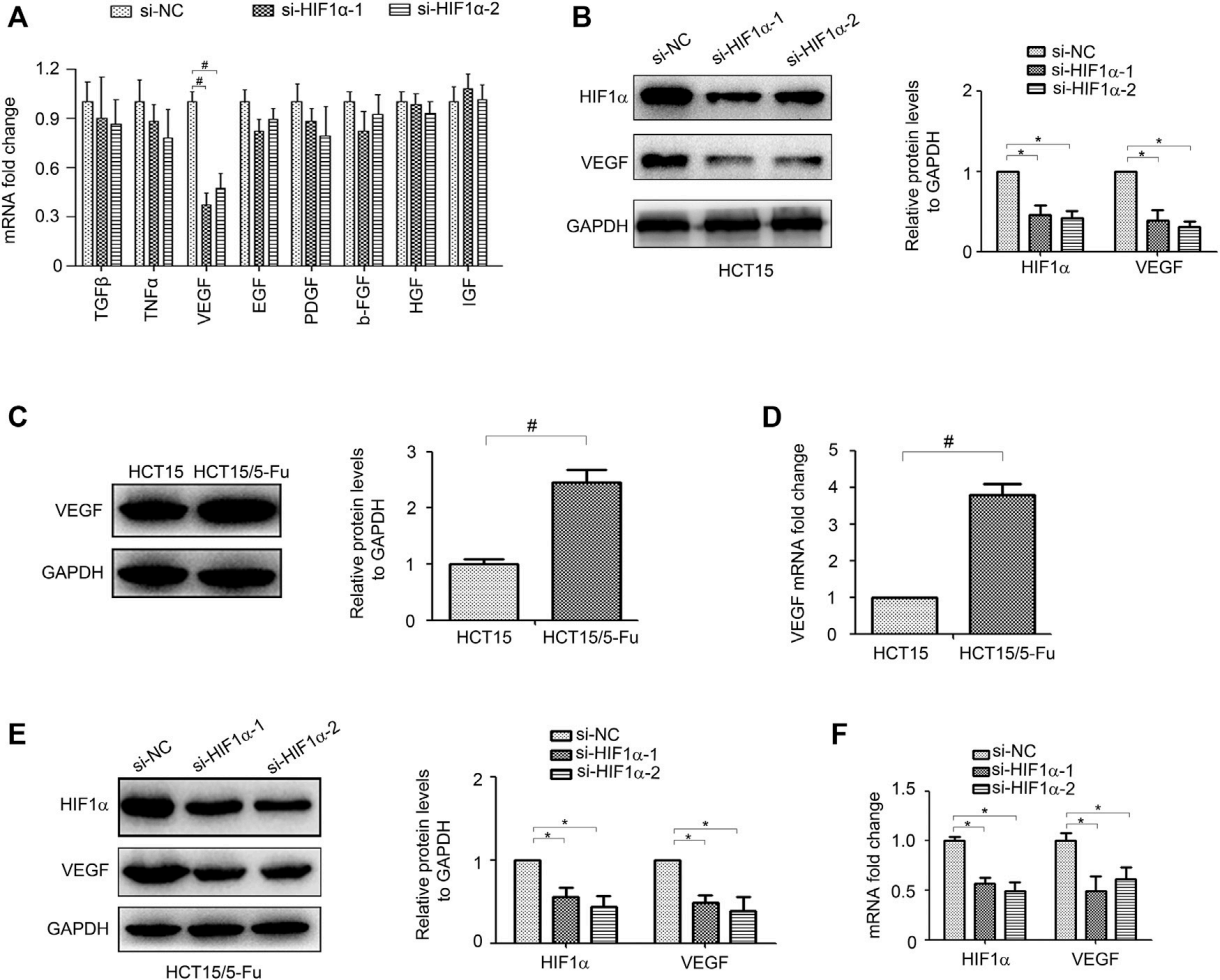
HIF1α regulates VEGF expression in CRC cells. **(A)** the mRNA expression of TGF-β, TNF-α, VEGF, EGF, PDGF, b-FGF, HGF, and IGF in HCT15 cells transfected with si-NC or si-HIF1α for 48 h were detected by RT-PCR. **(B)** the protein expression of VEGF in HCT15 cells transfected with si-NC or si-HIF1α for 48 h were detected by WB. **(C,D)** the mRNA and protein expression of VEGF in HCT15 and HCT15/5-Fu cells were detected by WB and RT-PCR, respectively. **(E,F)** the mRNA and protein expression of VEGF in HCT15/5-Fu cells transfected with si-NC or si-HIF1α for 48 h were detected by WB and RT-PCR, respectively. #: *p* < 0.01, *: *p* < 0.05.

The tumor microenvironment is increasingly identified as a crucial regulator to tumor progression (Spill et al., 2016; Ribeiro et al., 2020). Cancer cells promote their own progression by releasing multiple growth factors which interact with themselves directly through both autocrine and paracrine manners (Quail and Joyce, 2013). To gain further insight into how HIF1α induces CRC progression, we examined the effect of HIF1α on many microenvironmental genes, including TGF-β, TNF-α, VEGF, EGF, PDGF, b-FGF, HGF, and IGF, which have been implicated in promoting tumor development. Importantly, we found that knockdown of HIF1α significantly inhibited VEGF expression (Figures 4A,B). Moreover, VEGF was overexpressed in HCT15/5-Fu cells (Figures 4C,D). Knockdown of HIF1α also decreased VEGF expression in HCT15/5-Fu cells (Figures 4E,F).

### VEGF Plays an Important Role in HIF1α Mediated Aggressive Phenotype of 5-Fu Resistant HCT15 Cells

**FIGURE 5:**
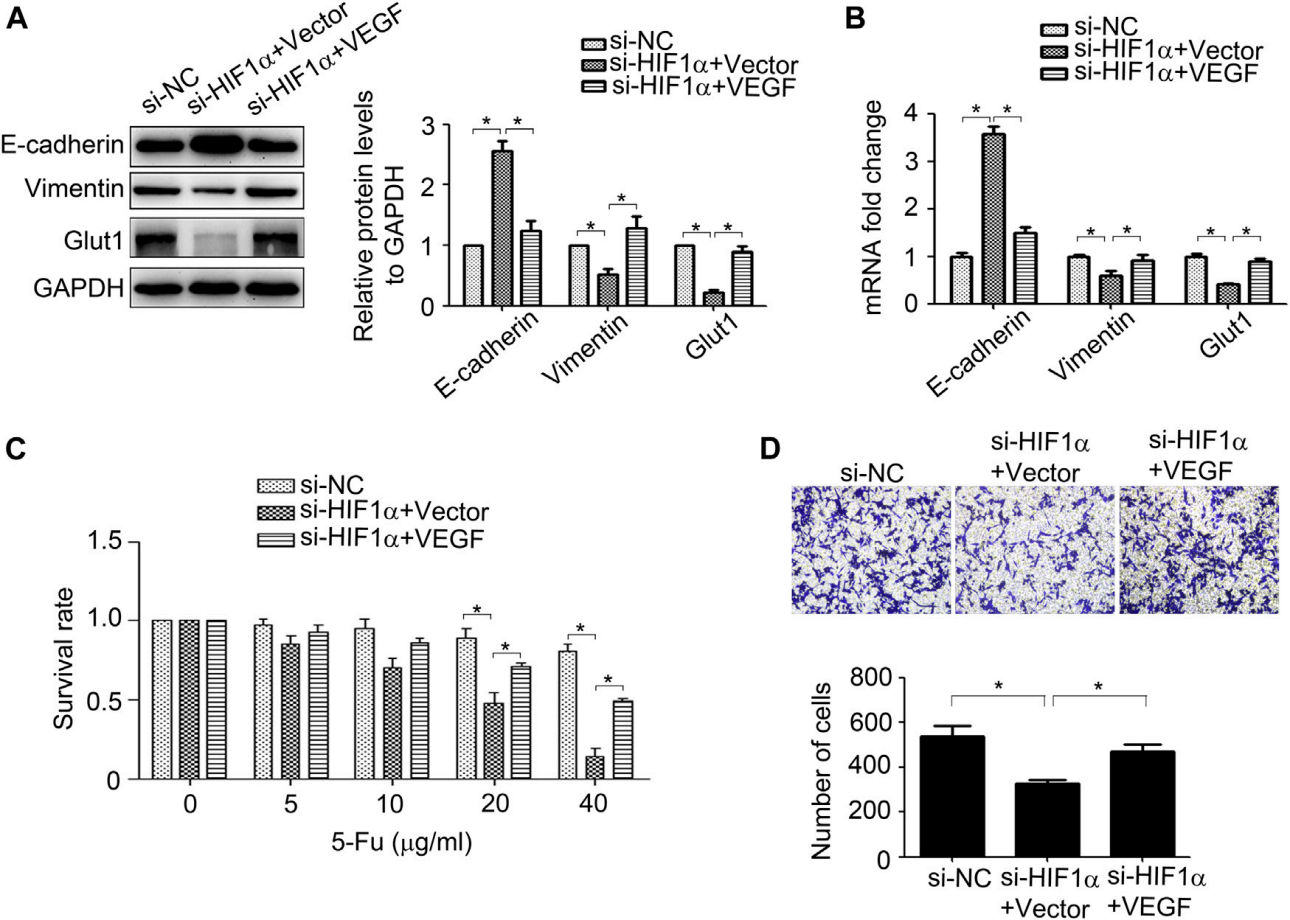
VEGF mediates HIF1α-maintained the aggressive phenotype of 5-Fu resistant CRC cells. **(A,B)** Expression of HIF1α, E-cadherin, Vimentin, and Glut1 protein and mRNA in HCT15/5-Fu cells transfected with si-NC, si-HIF1α+vector or si-HIF1α+VEGF for 48 h were detected byWB and RT-PCR, respectively. **(C)** HCT15/5-Fu cells transfected with si-NC, si-HIF1α+control vector or si-HIF1α+VEGF overexpression plasmid for 24 h were treated with increasing concentrations of 5-Fu for 48 h. CCK8 assay was used to quantify the viable cells. **(D)** HCT15/5-Fu cells were transfected with si-NC, si-HIF1α+vector or si-HIF1α+VEGF for 48 h, the migration capability was detected by transwell assay. Data represented as mean ± SD were from three independent experiments. *: *p* < 0.05.

Next, we investigate the roles of HIF1α and VEGF in regulating of CRC progression. As shown in Figures 5A,B, inhibition of HIF1α up-regulated E-cadherin expression, while down-regulated Vimentin and Glut1 expression in HCT15/5-Fu cells. Moreover, suppression of HIF1α enhanced the sensibility of drug resistant cells to 5-Fu and weakened its migration ability (Figures 5C,D). However, overexpression of VEGF partly eliminated the effect of HIF1α on E-cadherin, Vimentin, Glut1 expression and the malignant phenotype in HCT15/5-Fu cells (Figures 5A–D). These observations demonstrated that HIF1α/VEGF signal was essential for maintaining the aggressive phenotype of HCT15/5-Fu cells.

### AKT/GSK3β Signal Maintains the Aggressive Phenotype of 5-Fu Resistant HCT15 Cells

**FIGURE 6:**
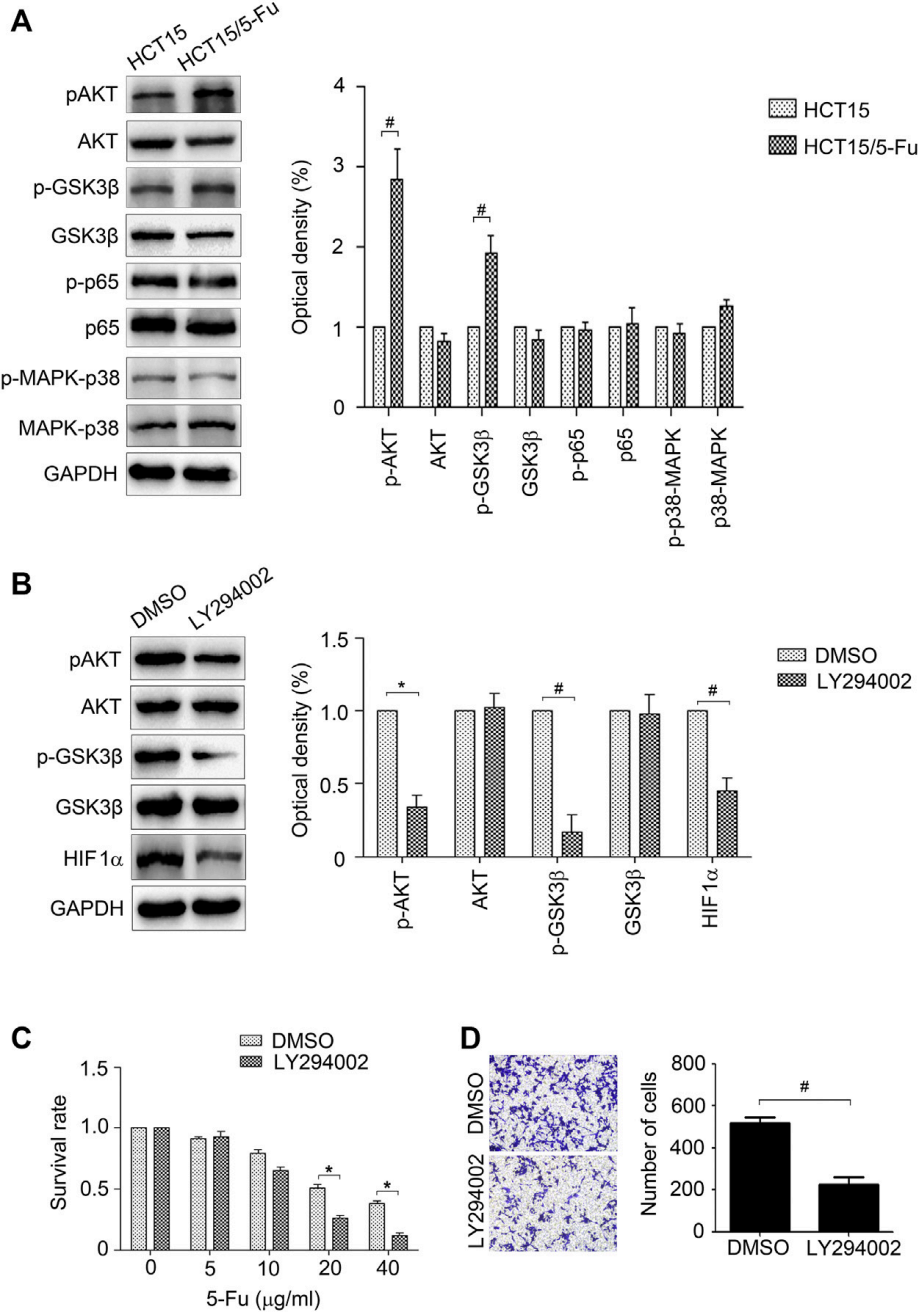
AKT/GSK3β signal is crucial for the aggressive phenotype of 5-Fu resistant CRC cells. **(A)** The expression of *p*-AKT, AKT, *p*-GSK3β, GSK3β, p-p65, p65, p-p38-MAPK and p38-MAPK in HCT15 and HCT15/5-Fu cells were detected by WB. **(B)** HCT15/5-Fu cells were treated with LY294002 (20 μM) for 24 h, the expression of *p*-AKT, AKT, *p*-GSK3β, GSK3β and HIF1α were examined by WB. **(C)** HCT15/5-Fu cells were treated with LY294002 (20 μM) or DMSO for 24 h, then treated with increasing concentrations of 5-Fu for 48 h. CCK8 assay was used to quantify the viable cells. **(D)** Transwell assay was performed to examine the migration of HCT15/5-Fu cells treated with LY294002 (20 μM) or DMSO for 24 h. Data represented as mean ± SD were from three independent experiments. *: *p* < 0.05, #: *p* < 0.01.

The AKT, p65, and p38-MAPK signals were measured for their crucial role in regulating EMT and drug resistance (Liang et al., 2019; Sale et al., 2019; Liang et al., 2020). The results of WB displayed that AKT signal was activated in HCT15/5-Fu cells, while the other signals have no obvious change (Figure 6A). The activity of GSK3β, an important kinase regulated by AKT signal, was also detected for its role in controlling HIF1α expression. As shown in Figure 5A, pGSK3β was increased in HCT15/5-Fu cells. LY294002, a PI3K/AKT signal inhibitor was used to prove the role of AKT/GSK3β pathway in HCT15/5-Fu cells. The results showed that LY294002 treatment significantly decreased pAKT, pGSK3β and HIF1α expression (Figure 6B). Furthermore, suppression of AKT/GSK3β reduced migration capability (Figure 6C) and enhanced 5-Fu sensitivity of HCT15/5-Fu cells (Figure 6D). Taken together, these results demonstrated that activated AKT/GSK3β signal contributed to the aggressive phenotype of HCT15/5-Fu cells.

### VEGF Feedback Regulates HIF1α Expression Through AKT/GSK3β Signal

**FIGURE 7:**
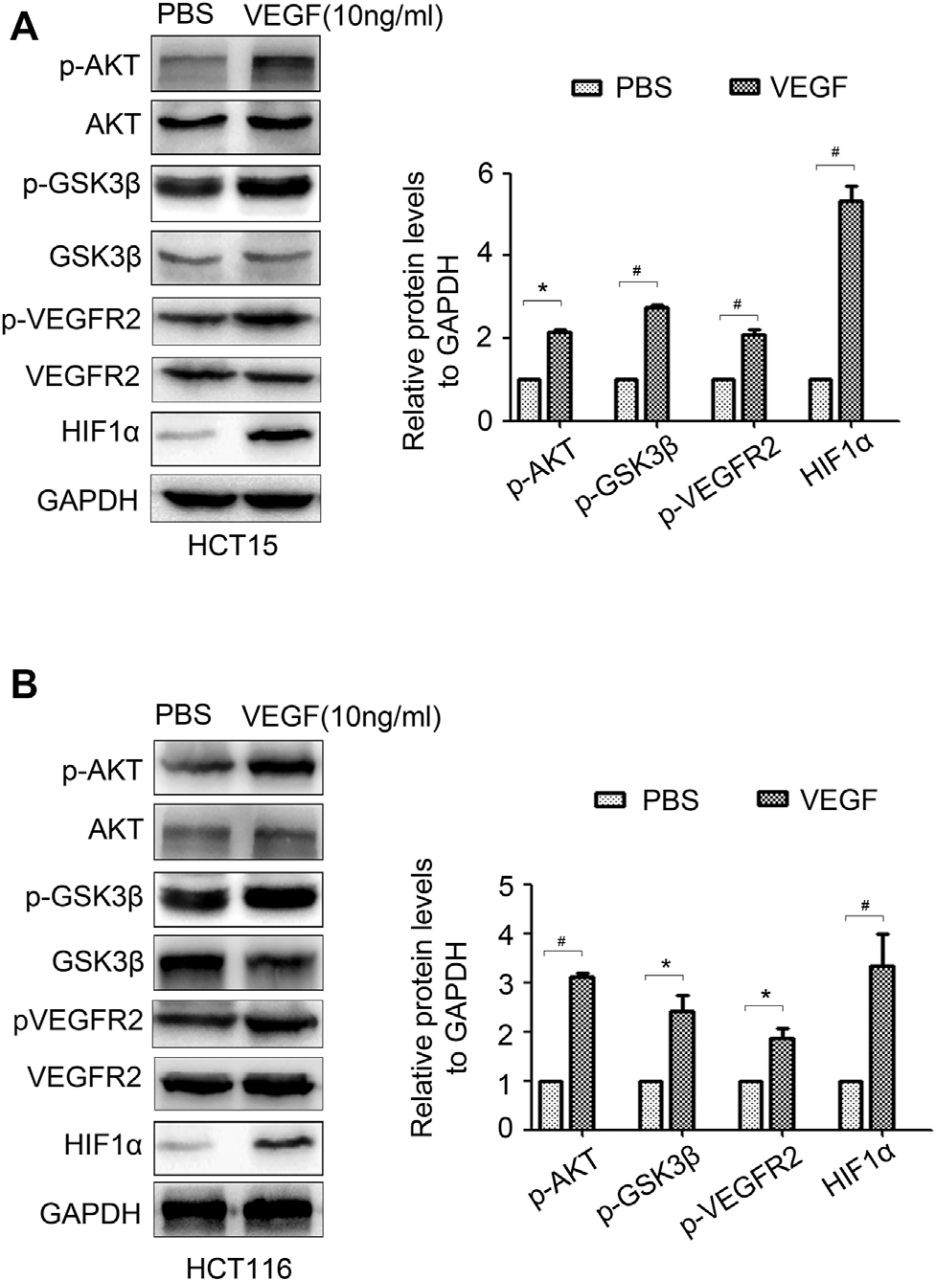
VEGF feedback regulates HIF1α expression in CRC cells. **(A,B)** The expression of *p*-AKT, AKT, *p*-GSK3β, GSK3β, *p*-VEGFR2, VEGFR2 and HIF1α in HCT15 and HCT116 cells treated with PBS or VEGF (10 ng/ml) for 24 h were detected by WB. *: *p* < 0.05, #: *p* < 0.01.

As an important inflammatory factor, VEGF can control tumor progression (Matsumoto and Ema, 2014). We wondered whether VEGF can feedback regulate HIF1α expression. VEGF was added in HCT15 and HCT116 cells, AKT/GSK3β signal and HIF1α expression were detected. The results showed that VEGF treatment significantly activated AKT/GSK3β signal and promoted HIF1α expression (Figures 7A,B). Moreover, VEGF treatment promoted phosphorylation of VEGFR2 which is a popular VEGF receptor expressed on many malignant cells and mediates the autocrine effect (Figures 7A,B). These results proved that VEGF could activate VEGFR and feedback regulate HIF1α expression via activating AKT/GSK3β signal.

### HIF1α/VEGF are High Expressed in CRC Tissues and Predict Poor Prognosis

**FIGURE 8:**
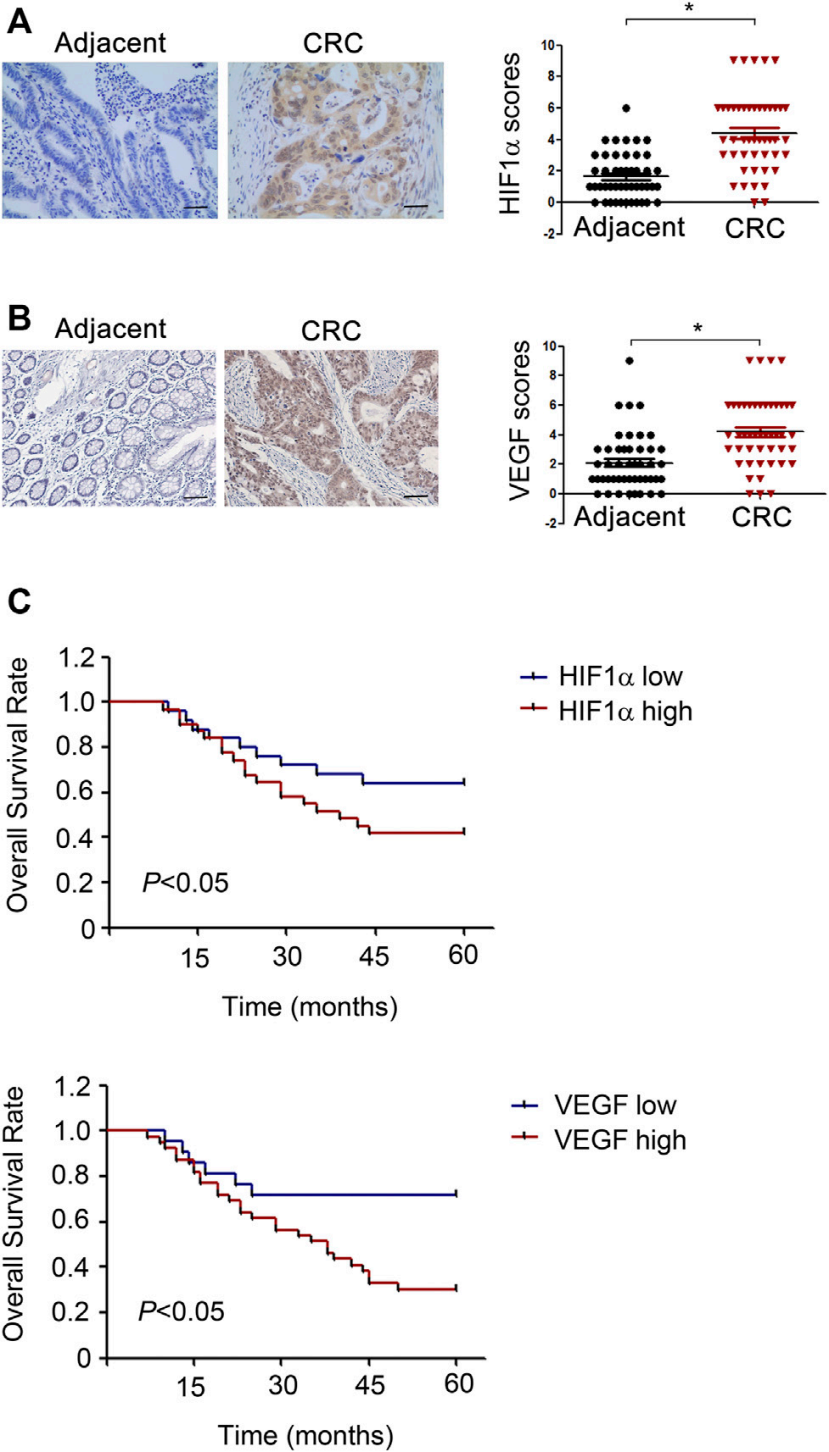
HIF1α and VEGF are high expressed in CRC patient tissues and predict poor prognosis. **(A,B)** Left, Representative IHC staining of HIF1α in CRC tumor sample and normal tissue sample. Right, Scores for HIF1α and VEGF staining in 60 pairs tumor tissue and adjacent normal tissue samples from CRC patients. **(C)** Univariate survival analysis of overall survival in CRC patients as determined by Kaplan-Meier plots estimates based on HIF1α and VEGF protein expression.

To validate the correlation of HIF1α, VEGF with prognosis of CRC patients, HIF1α and VEGF expressions in 60 pairs of CRC patient tissues were detected. As illustrated by IHC, tumor tissues displayed an increase expression of HIF1α and VEGF compared to the adjacent normal tissues (Figures 8A,B). Besides, CRC patients with increased HIF1α and VEGF expression exhibited reduced overall survival (Figure 8C). In conclusion, HIF1α and VEGF were high expressed in CRC patient tissues and predicted poor prognosis.

## Discussion

In recent years, the treatment of CRC advances by leaps and bounds. Surgical resection is the main way to treat CRC in clinic. However, the diagnosis of CRC is often delayed due to unclear and ambiguous symptoms, leading to rending the tumors non-resectable. Moreover, Patients with recurrent CRC after complete resection usually require palliative care. However, most patients have little or no benefit from adjuvant therapy, largely due to the development of drug resistance (Wei et al., 2019). Therefore, effective strategies are needed to address this problem. By screening the expression of drug resistant molecules, we identified the up-regulation of Glut1 in drug resistant cells. As was known, high rates of glucose metabolism provide ATP energy for cancer cells and plays an important role in drug resistant (Blondy et al., 2020). Glut1 controls the first step of glucose metabolism. High expression of Glut1 can protect cancer cells from oxidative stress induced by glucose deficiency and enhance anti-apoptotic activity (Gonzalez-Menendez et al., 2018). Since Glut1 is the key molecular involved in these pathways, its role in mediating resistance was investigated in this study. After the knockdown of Glut1, the sensitivity of 5-Fu resistant CRC cells was enhanced. These results indicated that up-regulation of Glut1 expression is responsible for maintaining the drug resistant phenotype.

Drug resistance is often accompanied with metastasis, which further neutralizes the therapeutic effect (Chakraborty et al., 2019; Siveen et al., 2019). Emerging studies have confirmed that drug resistant cells readily acquire EMT phenotype (Wei et al., 2020). Similarly, we found that drug resistant CRC cells displayed EMT features and enhanced HIF1α expression, which facilitating EMT conversion in various cancer cells. HIF1α affects the transcription of numerous genes, and directly stimulates the production of EMT transcription factors and affects the activity of EMT signaling pathways (Hapke and Haake, 2020). Inversely, knockdown of HIF1α inhibited migration capacity and promoted MET conversion. A set of transcription factors, including Snail, Slug, ZEB1, Twist, play a crucial role in regulation of EMT and drug resistance (Hsu et al., 2010; Xiao et al., 2018; Sale et al., 2019; Yochum et al., 2019), no obviously up-regulation of them was detected in resistant CRC cells. Besides EMT, HIF1α induces the drug resistance in numerous cancer cells. HIF1α promoted drug resistance in human medulloblastoma by inhibition of CYP2B6, CYP3A4 and CYP3A5 expression, which in turn resulted in decreased conversion of CPA and IFA into their active forms and thus to diminished cytotoxicity (Valencia-Cervantes et al., 2019). In CRC cells, suppression of HIF1α reversed multi-drug resistance by down-regulating the expression of MDR1. Consistent to these findings, our date showed that knockdown of HIF1α enhanced drug sensitivity of 5-Fu resistant CRC cells and reduced Glut1 expression, the downstream of hypoxia and HIF1α.

Proinflammatory factors in the inflammatory microenvironment plays an important role in tumor metastasis and chemoresistance. Our study provides evidence that the inhibitory effect of HIF1α knockdown on aggressive phenotype could be partially regained by VEGF overexpression. VEGF, secreted primarily by macrophages, is one of the major inducers of angiogenesis and is tightly correlated with tumor progression and metastasis. A previous study demonstrated that HIF1α stabilized by Lysine-specific demethylase 1 cooperates with CBP and MTA1 to enhance VEGF induced tumor angiogenesis (Lee et al., 2017). Similarly, we also found that suppression of HIF1α significantly decreases VEGF expression. Moreover, we observed that VEGF mediated HIF1α-induced aggressive phenotype of 5-Fu resistant CRC cells. High levels of HIF1α and VEGF were correlated with shorter survival in CRC patients. Thus, our results indicated that HIF1α is a positive regulator of VEGF and that dysregulated HIF1α/VEGF signaling contributes to metastasis and 5-Fu resistance of CRC.

In our attempt to investigate the signaling pathways which mediated EMT and drug resistance, we discovered the AKT signal was activated in 5-Fu resistant CRC cells. Although NF-κB, and p38-MAPK pathways play important roles in regulating EMT and drug resistance, no significant changes of them were observed in this study. A previous study proved that AKT maintained EMT phenotype in the gefitinib resistant head and neck squamous cell carcinoma cells by regulating of Snail expression (Maseki et al., 2012). Recently, a study revealed that the AKT signal-mediated Slug expression led to oxaliplatin resistance in CRC *via* up-regulation of ERCC1 (Wei et al., 2020). Consistently, we found that suppression of AKT pathway significantly inhibited HIF1α expression and reduced drug resistance. The expression and location of HIF1α can be regulated by GSK3β (Cheng et al., 2014), an important kinase downstream of AKT. Our results showed that GSK3β activity was dramatically decreased in 5-Fu resistant CRC cells and inhibition of AKT pathway enhanced GSK3β activity thus decreased HIF1α expression. A recent study showed that VEGF-mediated transcriptional induction of HIF1α enhanced chemoresistance and reduced cell apoptosis (Jiang et al., 2021). Here, we revealed that VEGF could feedback regulate HIF1α expression through activating AKT/GSK3β signaling.

**FIGURE 9:**
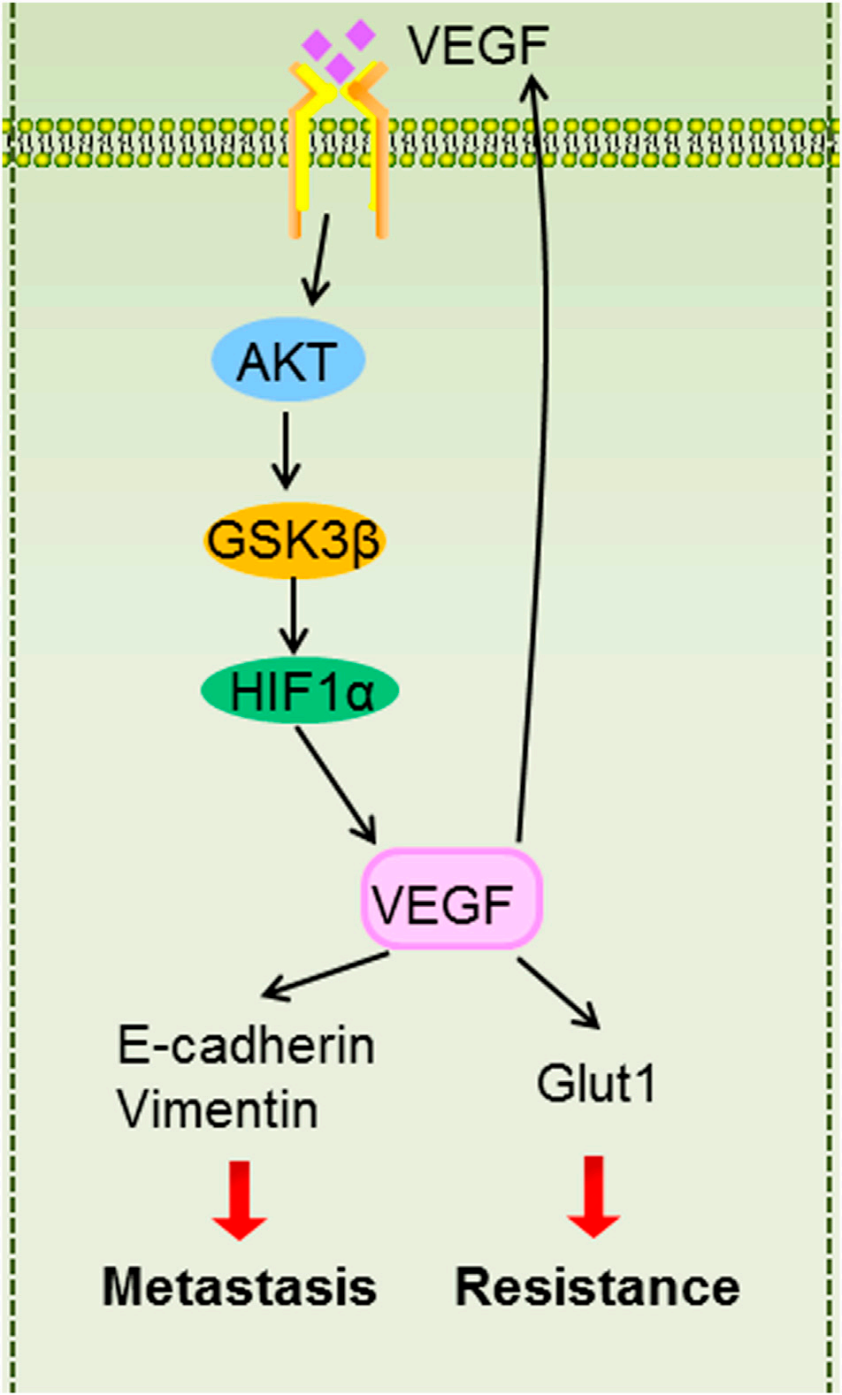
The graphic illustration of HIF1α/VEGF feedback loop contributes to 5-Fu resistance and metastasis in CRC.

In summary, our findings suggest that inflammatory microenvironment induced by hypoxia mediated metastasis and 5-Fu resistance. Mechanistically, HIF1α/VEGF feedback loop maintains metastasis and resistant phenotype by regulating EMT and Glut1. AKT/GSK3β signaling is involved in controlling HIF1α/VEGF feedback loop regulation (Figure 9). Our study highlights the potential of targeting HIF1α/VEGF as a novel therapeutic strategy.

## Data Availability

The raw data supporting the conclusion of this article will be made available by the authors, without undue reservation.
